# Microplastic Contamination of Non-Mulched Agricultural Soils in Bangladesh: Detection, Characterization, Source Apportionment and Probabilistic Health Risk Assessment

**DOI:** 10.3390/jox14020046

**Published:** 2024-06-19

**Authors:** Sumaya Sharmin, Qingyue Wang, Md. Rezwanul Islam, Weiqian Wang, Christian Ebere Enyoh

**Affiliations:** 1 Graduate School of Science and Engineering, Saitama University, 255 Shimo-Okubo, Sakura-ku, Saitama 338-8570, Japan; islam.m.r.282@ms.saitama-u.ac.jp (M.R.I.); weiqian@mail.saitama-u.ac.jp (W.W.); enyoh.c.e.527@ms.saitama-u.ac.jp (C.E.E.); 2 Department of Agricultural Extension, Khamarbari, Dhaka 1215, Bangladesh

**Keywords:** microplastics, non-mulched soil, source apportionment, health hazard, soil layer

## Abstract

Microplastic contamination in agricultural soil is an emerging problem worldwide as it contaminates the food chain. Therefore, this research investigated the distribution of microplastics (MPs) in agricultural soils without mulch at various depths (0–5, 5–10, and 10–15 cm) across different zones: rural, local market, industrial, coastal, and research areas. The detection of MP types and morphology was conducted using FTIR and fluorescence microscopy, respectively. Eight types of MPs were identified, including high-density polyethylene (HDPE), low-density polyethylene (LDPE), polypropylene (PP), polyethylene terephthalate (PET), polyvinyl chloride (PVC), polyvinyl fluoride (PVF), polyvinyl alcohol (PVA), and polytetrafluoroethylene (PTFE), with concentrations ranging from 0.6 ± 0.21 to 3.71 ± 2.36 MPs/g of soil. The study found no significant trends in MP concentration, with ranges of 0–2.1 ± 0.38, 0–2.87 ± 0.55, and 0–2.0 ± 0.34 MPs/g of soil at depths of 0–5 cm, 5–10 cm, and 10–15 cm, respectively. The highest MP quantity was recorded at 8.67 in coastal area, while the lowest was 6.44 in the local market area. Various MP shapes, e.g., fiber, film, pellet, fragment, and irregular, were observed across all layers. PCA suggested irrigation and organic manure as potential sources of MPs. The estimated concentrations of MPs possessed low non-carcinogenic and carcinogenic risks to the farming community of Bangladesh.

## 1. Introduction

Microplastics (MPs) have emerged as a global concern, drawing attention to their association with health issues, including stillbirth, prematurity, birth defects, neuro disorders, allergies, asthma, respiratory diseases, and lung cancers, attributable to the toxic, mutagenic, and carcinogenic nature of MPs [1,2,3]. MPs enter the human body through multiple pathways, such as dermal contact, inhalation, and ingestion [4,5,6,7]. In modern agriculture, plastic utilization introduces MPs into soils through diverse practices, including plastic mulching, sewage irrigation, soil amendment, fertilizer coatings, littering, runoff, and atmospheric deposition [8,9,10,11,12,13,14,15]. The ecotoxicological effects of MPs on soil organisms encompass a range of potential impacts, including altered behavior, reduced reproductive success, and physiological disturbances, with implications for soil health and ecosystem functioning [16]. Moreover, the farming community is affected by MPs during farming practices and through the food chain. Farming communities in developing countries like Bangladesh are particularly susceptible to MP exposure due to adopting inadequate safety measures during the farming process.

Plastic films are indeed a significant contributor to microplastic pollution in agricultural soils. In Europe, a substantial amount of plastic, totaling 54 million tons, is utilized annually, with approximately 4% allocated for agricultural, farming, and gardening purposes [17]. Specifically, in Europe, 0.083 million tons of plastic are employed for mulch film in farming activities [18]. Conversely, in countries such as China, Japan, and South Korea, an estimated 700,000 tons of high-density polyethylene (HDPE) are utilized each year [19]. However, it is worth noting that this is not a significant source of microplastics in developing countries like Bangladesh, where the majority of farmers do not use plastic film as mulch. Very few progressive farmers and commercial agricultural farms are using plastic film as early adopters. Though the farmers are not using plastic film, they utilize various agricultural inputs such as compost [20], livestock manure (from chickens, cows, goats, and pigs), bacterial residues [21], chemical fertilizers [22], and other materials. Additionally, factors like irrigation pipes and agricultural equipment [23], coupled with precipitation [24], persist as potential sources of microplastics (MPs) in non-mulched agricultural soils. However, research on MP contamination in soil is concentrated mainly on mulched agricultural lands. Research on MP contamination in non-mulched agricultural fields is scarce. Furthermore, there is no research addressing MP contamination of agricultural fields with diversified geographic locations like coastal agricultural land, industrial adjacent agricultural land, research field areas, rural areas, and local market areas. However, few existing studies have been conducted on vegetable fields, uplands, and other agricultural soils [24,25,26]. In Bangladesh, this would be the baseline study for strata-wise detection of MPs in non-mulched agricultural soil.

Considering the research gaps, this study investigated MP contamination at different soil depths (0–5 cm, 5–10 cm, and 10–15 cm) at five distinct geographic locations. The objectives of the study include quantifying and identifying MPs in non-mulched agricultural soils, their distribution, shapes, and potential sources, evaluating health risks to farmers, and proposing effective solutions for mitigating microplastic pollution in agricultural lands. This research endeavors to provide comprehensive insights that are indispensable for safeguarding both agricultural productivity and human health in the face of the growing challenges posed by microplastics.

## 2. Materials and Methods

### 2.1. Soil Sample Collection

**Figure 1:**
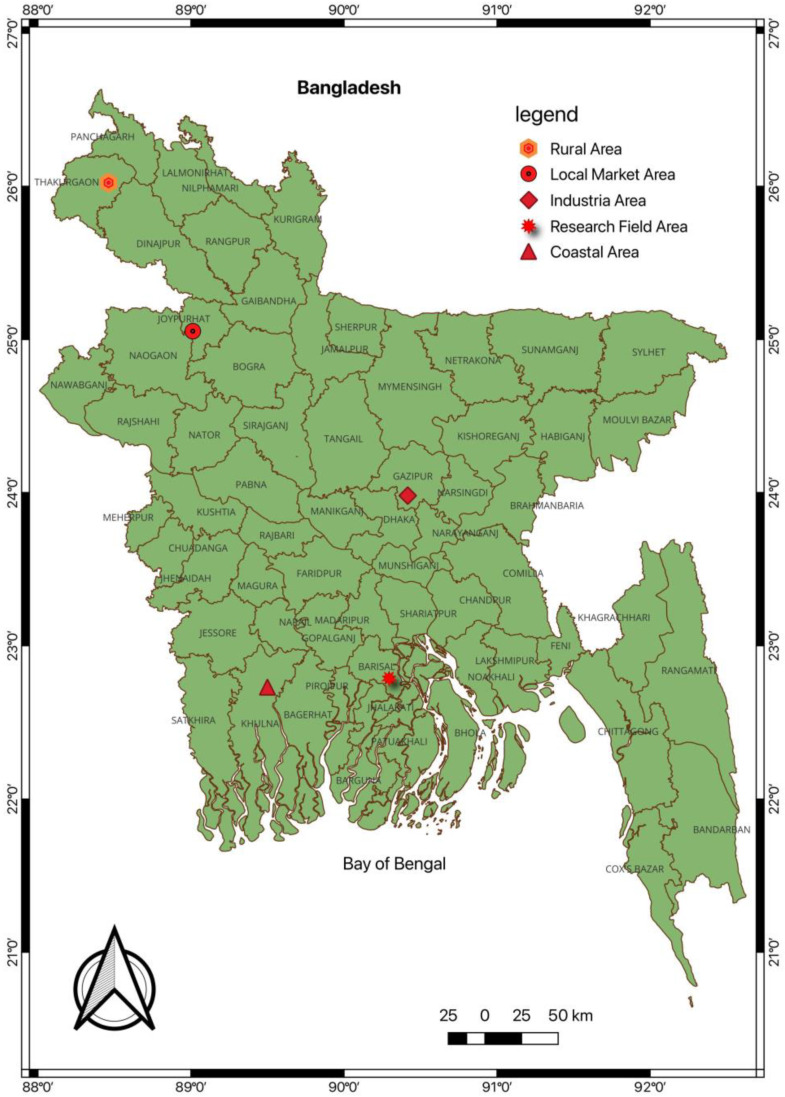
Soil sampling sites on the map of Bangladesh.

Soil was collected from five distinct agricultural areas devoid of plastic mulch film including rural, local market, industrial, coastal, and research areas across Bangladesh for the determination of MP contamination, as shown in Figure 1.

The sampling was performed from fields withstanding crops, using an auger to obtain undisturbed soil at depths of 0–5 cm, 5–10 cm, and 10–15 cm. Following a random sampling technique at five locations within each area ensured representative sampling, with similar samples combined to form composite samples. The general features of the selected areas are outlined in Table 1.

### 2.2. Soil Sample Preparation

Following collection, soil samples were cleaned properly to remove plant roots and other debris. Then, the soil samples were dried under shed conditions. The samples were then ground and sieved (1 mm) and stored in aluminum foil for further analysis. 

### 2.3. Soil Sample Pretreatment

Pretreatment involved density separation techniques, wherein 20 mL of 30% H_2_O_2_ was added to digest organic matter for 12 h, followed by filtration to remove H_2_O_2_ and the addition of 20 mL of NaCl (1.2 g/cm^3^) to facilitate flotation of low-density plastic particles. The floated particles were subsequently filtered for further analysis. For pretreatment, 1 g of dry soil was used.

### 2.4. Characterization

Morphological analysis was performed using a fluorescence microscope (MX6300, Meiji Techno Co., Tokyo, Japan). Pixera IN Studio software (version 3.5.2) was used to analyze the captured images. To detect the type of MPs, functional groups were measured using Fourier transform infrared (FTIR) spectroscopy (IR-6100, JASCO Co., Ltd., Hachioji, Tokyo, Japan).

### 2.5. Assessment of the Health Risk

#### 2.5.1. MP Risk Indices

Risk indices of microplastics (MPs) were determined for the soil samples using equations described by [29], with hazard scores based on toxicity levels as published by [26]: PP = 1, PET = 4, HDPE = 11, LDPE = 11, and PVC = 10001. The equation as follows:

(1) $$Polymar\;risk\;indices\;(pRi)=\sum \left(\frac{Number\;of\;individual\;MPs\;(pmi)}{Total\;MPs\;(pT)}\times Hazard\;score\;(Sj)\right)$$

(2) $$pRarea={\left(pR1\times pR2\times pR3\times \ldots \ldots \ldots \ldots \times pRn\right)}^{1}/n.$$

#### 2.5.2. Average Estimated Daily Intake (EDI) of MPs

The computation of MP contamination risk for individual farming people during farming activities, through ingestion, inhalation, and dermal pathways, was conducted. Measurement of the average estimated daily intake (EDI) of MPs utilized Equations (1)–(3), as per [30]. The parameters essential for these equations are specified in Table 2.

(3) $$Average\;daily\;intake\;\left(Ingestion\right)\;\left(\frac{\frac{mg}{Kg}}{day}\right):D_{Ingestion}=MP\times \frac{ED\times EF\times IngR}{AT\times BW}\;\times \;10^{-6}$$

(4) $$Average\;daily\;intake\;\left(Inhalation\right)\left(\frac{\frac{mg}{Kg}}{day}\right):D_{Inhalation}=MP\times \frac{ED\times EF\times InhR}{AT\times BW\times PEF}$$

(5) $$Average\;daily\;intake\;\left(Dermal\right)\left(\frac{\frac{mg}{Kg}}{day}\right):D_{Dermal}=MP\times \frac{ED\times EF\times SL\times SA\times ABS}{AT\times BW}\;\times \;10^{-6}$$

#### 2.5.3. Cancer Risk Assessment of MP

The assessment of cancer risk involved the consideration of the lifetime average daily dose (LADD) and cancer slope factor [30]. LADD values were determined using Equations (6)–(8) [35,36,37]. Cancer risk is influenced by the cancer slope factor (CSF), which measures the risk of cancer over a lifetime due to exposure to the carcinogenic agent. The CSF values for HDPE, LDPE, PET, PP, and PVC were 1.02, 1.02, 1.02, 0.24, and 1.9, respectively [38].

(6) $${LADD}_{Ingestion}=C\times \frac{EF}{AT}\times (\frac{{IngR}_{Child}\times {ED}_{CHild}}{{BW}_{Child}}+\frac{{IngR}_{Adu;t}\times {ED}_{Adult}}{{BW}_{Adult}})\;\times \;10^{-6}$$

(7) $${LADD}_{Inhalation}=C\times \frac{EF}{AT\times PEF}\times \left(\frac{{InhR}_{Child}\times {ED}_{CHild}}{{BW}_{Child}}+\frac{{InhR}_{Adu;t}\times {ED}_{Adult}}{{BW}_{Adult}}\right)$$

(8) $${LADD}_{Dermal}=C\times \frac{EF\times SA\times SL\times ABS}{AT}\times (\frac{{ED}_{CHild}}{{BW}_{Child}}+\frac{{ED}_{Adult}}{{BW}_{Adult}})\;\times \;10^{-6}$$

(9) $${CR}_{Ingestion}\;{=LADD}_{Ingestion}{\;\times \;CSF}_{Ingestion}$$

(10) $${CR}_{Inhalation}\;{=LADD}_{Inhalation}{\;\times \;CSF}_{Inhalation}$$

(11) $${CR}_{Dermal}\;{=LADD}_{Dermal}{\;\times \;CSF}_{Dermal}$$

(12) $${CCR=\sum CR=CR}_{Ingestion}{\;+\;CR}_{Inhalation}{\;+\;CR}_{Dermal}$$

### 2.6. Quality Control

An iron auger was used to avoid contamination of MPs. Samples underwent processing (cleaning, drying, and crushing) in a plastic-free environment. The samples were stored in aluminum foil. The apparatus used for analysis was devoid of plastic, having been thoroughly cleaned, rinsed, sonicated, dried in an oven, and sealed with aluminum foil to prevent any further contamination. Ultrapure (Type 1) water was utilized to prepare the NaCl solution and clean the apparatus. Care was taken to prevent contamination throughout sieving, digestion, density separation, filtration, transportation, characterization, and identification. Cotton aprons and gloves were worn to prevent contamination. Samples were tested in triplicate to ensure data accuracy. Blank sampling (trip blank) was considered during the study. It is processed and analyzed using identical methods to the actual samples, including steps like sieving, digestion, density separation, and spectroscopy.

### 2.7. Statistical Analysis

Statistical analysis was conducted using IBM SPSS Statistics 20 and Microsoft Office Excel 2013, with differences between means assessed using Duncan’s multiple range test (DMRT).

## 3. Results and Discussion

### 3.1. Morphology of the Particles

**Figure 2:**
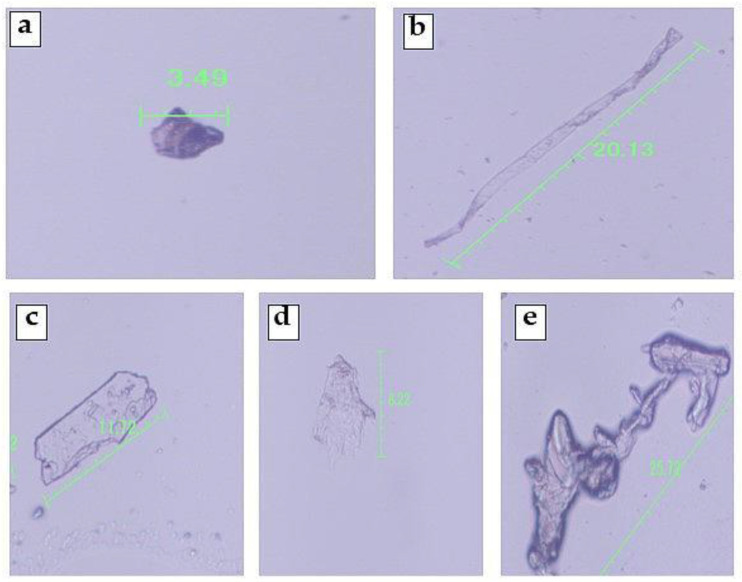
Different shapes of the MPs detected under fluorescence microscope. (**a**) Pellet; (**b**) fiber; (**c**) fragment; (**d**) film; (**e**) irregular.

The study revealed various sizes and forms of MPs, which are mentioned in Figure 2. The shape was comprised of fibers, films, pellets, fragments, and irregular shapes. MP enters soil by different means including through compost, sewage, mulching, wastewater, etc. [39,40,41]. Agricultural practices such as plowing and cultivation inadvertently contribute to the breakdown of larger plastic particles in soil, while environmental factors such as sunlight and moisture exacerbate the presence of fragment-shaped microplastics [42]. As larger plastic debris gradually degrades into smaller particles over time, pellet-shaped MPs become prevalent in soil [43]. Fibers, primarily detected in agricultural soils in Bangladesh, originate from diverse sources like synthetic textiles and plastics, raising environmental concerns due to their widespread dispersion [44]. These fibers infiltrate agricultural soil through pathways such as irrigation water, wind deposition, and erosion, potentially affecting soil health and ecosystems. Film-shaped microplastics, resembling thin sheets, enter agricultural soil through the composting of plastic bags and wrappings. Over time, exposure to sunlight, temperature fluctuations, and agricultural activities cause these plastics to fragment into smaller pieces, including film-shaped microplastics, which become integrated into the soil [45]. Irregular shaped microplastics stem from the degradation of plastic waste resulting from improper disposal practices such as open dumping or burning, further complicating the issue. Various researchers [41,46,47,48,49] have reported different shapes of microplastics found in agricultural soils across various regions.

### 3.2. Detection of Types of MPs by FTIR

**Figure 3:**
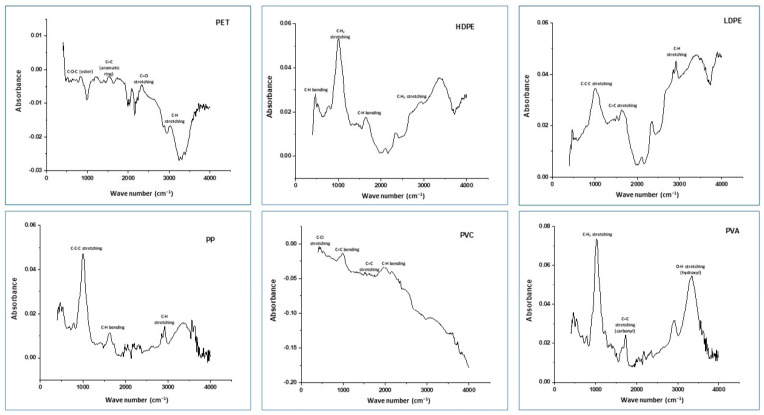
FTIR spectra of the detected particles.

The infrared spectrum provides valuable insights into the structural composition of various polymers and their functional groups, as outlined in Figure 3. A distinct peak between 1100 and 1000 cm⁻^1^, indicating C–O–C bond stretching, alongside peaks at 1500–1600 cm⁻^1^, representing its aromatic ring structure, and peaks at 1700–1725 cm⁻^1^ for carbonyl groups (C=O) in the ester linkage translate to PET MPs [50]. Additionally, peaks near 1000 cm⁻^1^, denoting CH_2_ group stretching, with additional peaks at 1600–1700 cm⁻^1^ for C–H bending, and strong peaks at 2850–2917 cm⁻^1^, reflecting C–H bond stretching in the polymer backbone, indicate the presence of HDPE MPs [51]. Peaks around 1150–1000 cm⁻^1^ for C–C–C bond stretching, accompanied by peaks between 1600 and 1700 cm⁻^1^ for C–H bending, and peaks at 2800–3000 cm⁻^1^ for C–H bond stretching in the polymer backbone reveal PE MPs. C–H stretching vibrations observed between 3000 and 2800 cm⁻^1^, primarily composed of carbon–carbon (C–C) bonds with broad peaks around 1000 cm⁻^1^, show LDPE MPs. Additionally, distinctive peaks around 600–800 cm⁻^1^ indicate C–Cl stretching vibrations for polyvinyl chloride (PVC) [52], while characteristic peaks at 1750–1730 cm⁻^1^ for C=O stretching, broad peaks at 3200–3500 cm⁻^1^ for O–H stretching, and peaks at 1450–1375 cm⁻^1^ for CH_2_ bending vibrations show PVA MPs. The authors of [53] also reported C–H symmetrical stretching, deformation of C–O, and aromatic rings at 2908 cm⁻^1^, 1342 cm⁻^1^, 1410 cm^−1^, 1453 cm^−1^, and 972 cm^−1^, respectively, mentioning the presence of PET microplastics. The authors of [54] also illustrated the FTIR spectrum of PP, which peaks at 2923.27, 2838.67, and 1631.29 cm^−1^.

### 3.3. Relative Distribution of Different MPs in Non-Mulched Soils

**Figure 4:**
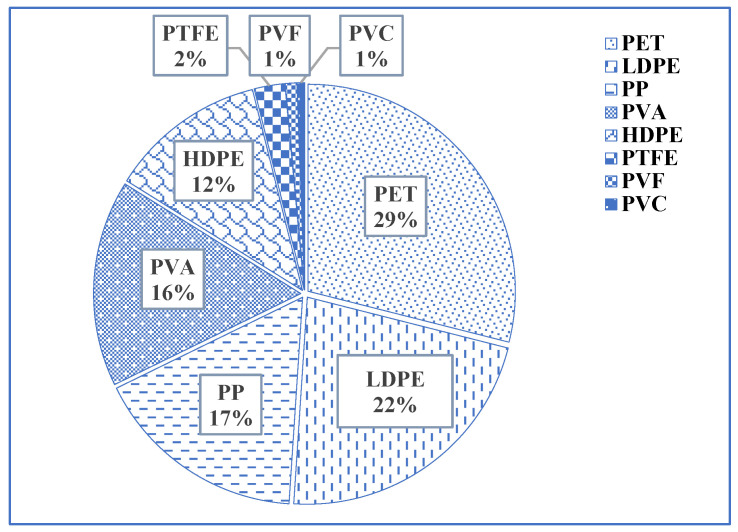
Relative distribution of different MPs in non-mulched soils.

Figure 4 outlines eight distinct types of plastics and their respective proportions in the studied soil. Among these plastics, PET constitutes the largest proportion, accounting for approximately 28.96% of the total composition. Following PET, LDPE represents around 22.09%, and PP contributes approximately 16.72%. The PVA holds a similar share, accounting for approximately 16.12%. HDPE follows with around 12.24%, while PTFE constitutes approximately 2.39%. The PVF and PVC make up smaller percentages, approximately 0.90% and 0.60%, respectively. These plastic materials enter soil from applied compost, littering, improper disposal of plastic waste, agricultural activities, etc. PVC derivatives like PVA were much higher in the reported soil. Basically, more irrigation water needs to be applied to non-mulched agricultural soil than to mulched agricultural soil. PVC pipes are generally used to supply irrigation water, which could be a reason for more PVA content in the reported soils. The findings align with a study conducted in Dhaka, Bangladesh, by [44], confirming the presence of HDPE, PVC, PP, and LDPE in soil and sludge. The authors of [55] also reported LDPE and HDPE in different organic fertilizer sources.

### 3.4. Area-Based Distribution of MPs in Non-Mulched Agricultural Soils

**Figure 5:**
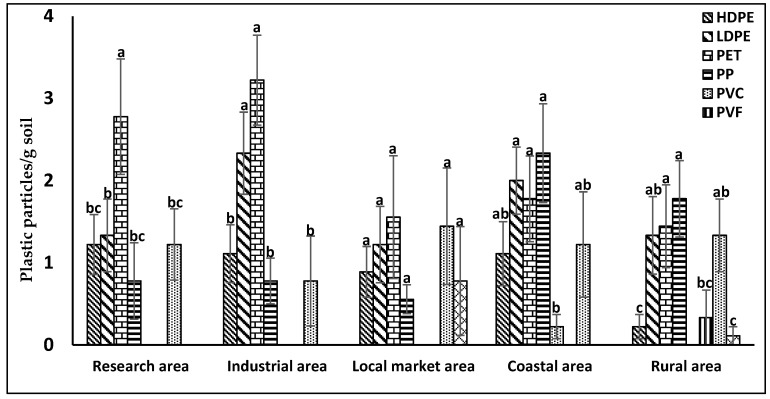
Area-based distribution of different microplastic particles in Bangladesh. Significant differences (*p* < 0.05) exist among mean values associated with bars marked with uncommon letters.

The results offer a quantitative understanding of the distribution and relative significance of various plastic types across diverse geographic locations. Figure 5 displays MP particle counts ranging from 6.44 to 8.67 per gram of soil, with statistically significant differences. The highest quantity, 8.67, was recorded in field soils of the coastal area (Khulna), while the lowest (6.44) was observed in the local market area (Joypurhat). Among the different types of MPs, PET and LDPE exhibited higher levels in the industrial area, potentially stemming from improper disposal of polythene and PET, ultimately infiltrating agricultural soils. In the research area, PET was more prevalent compared to other plastics, likely due to organic matter containing elevated concentrations of plastic particles. In the industrial area, issues such as overpopulation and inadequate waste management contribute to the increased presence of PET and LDPE. Conversely, PP, LDPE, and PET show heightened significance in coastal areas, possibly reflecting their sources from organic matter. PVC and PVF were solely recorded in coastal and rural areas, potentially originating from irrigation channels. The authors of [44] also noted the presence of MPs in soil near industrial areas of Savar, Bangladesh. Additionally, [55] found 1529.62 ± 420.2 MP particles/kg of organic manures.

### 3.5. Strata-Wise MP Distribution in Non-Mulched Agricultural Soil

**Figure 6:**
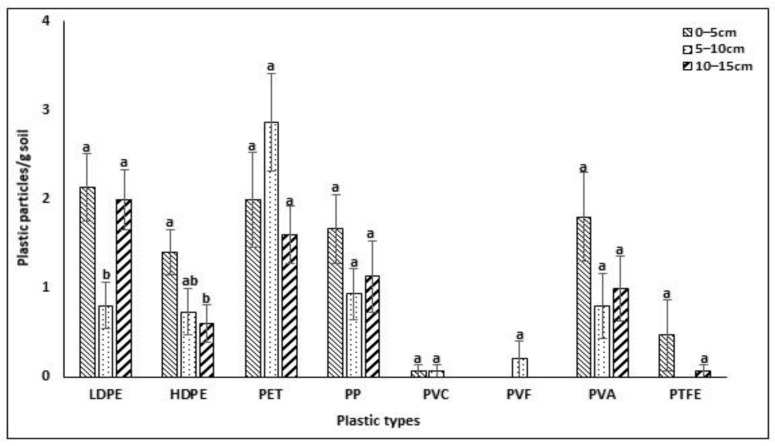
Strata-wise distribution of different microplastic particles in Bangladesh. Significant differences (*p* < 0.05) exist among mean values associated with bars marked with uncommon letters.

The figure presents the distribution of microplastics across various types of plastic polymers within different soil depths. It categorizes microplastics into three soil depths, each with corresponding concentrations measured as the number of particles per gram of soil. The detection of strata-based MPs is important as crop roots are distributed at different soil depths. The results revealed that microplastics were present at three different soil depths and differed statistically (*p* < 0.05), ranging from 0 to 2.87 ± 0.55 plastic particles/g soil (Figure 6). Within the top layer (0–5 cm), LDPE (low-density polyethylene) exhibits the highest concentration (2.13), followed by PET (polyethylene terephthalate) (2.00). As we move deeper into the soil, the distribution varies. For instance, within the 5–10 cm layer, PET becomes dominant with a concentration of 2.87, while LDPE decreases to 0.8. Similarly, at a depth of 10–15 cm, HDPE (high-density polyethylene) shows a significant decrease compared to the previous layers, whereas PET maintains a relatively stable concentration. The data suggest variations in microplastic distribution across different soil depths, indicating potential factors influencing the deposition and degradation of plastic polymers within soil profiles. The authors of [25] identified PP, PA, and PS at the 0–5 cm depth of agricultural soil, whereas [41] reported PP and PVC at the 0–30 cm depth of soil.

### 3.6. Source Apportionment of the Detected MPs

**Figure 7:**
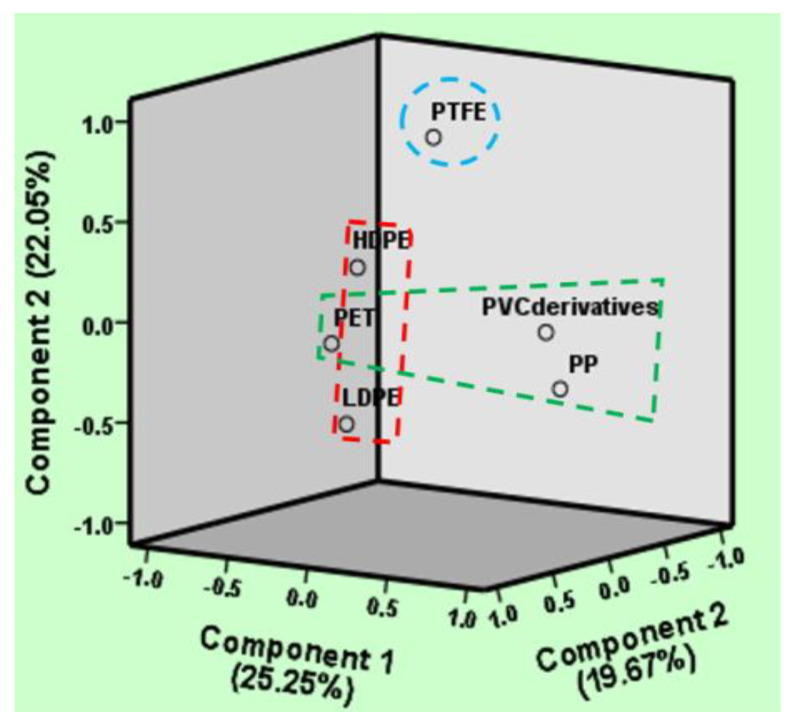
Principal component analysis of the detected MPs.

The results revealed three primary sources identified from non-mulched agricultural soil through principal component analysis (PCA): component 1 (irrigation sources) encompasses derivatives of PET, PP, and PVC mentioned in Figure 7. Farmers commonly employ water from surface reservoirs, mainly rivers, for field irrigation, and the extensive use of PVC pipes for water conveyance presents another potential avenue for microplastic introduction. This observation is corroborated by reports indicating the presence of PP and PVC in soils subjected to sewage and wastewater irrigation. The authors of [56] reported the presence of MPs originating from PVC pipes during irrigation. Component 2 (organic manure) involving LDPE and HDPE indicates compost as a significant contributor to microplastic presence in agricultural soil, particularly within the Bangladeshi context, aligning with findings identifying PE presence originating from organic manure. Component 3 (others) includes PTFE, found in various industrial applications such as the manufacturing of agricultural equipment and machinery. In Bangladesh, agricultural soils may face potential contamination with PTFE from machinery runoff, equipment cleaning activities, or material breakdown within the agricultural sector that incorporates PTFE.

### 3.7. Health Risk Assessment

#### 3.7.1. MP Polymer Risk Indices (pRi)

**Figure 8:**
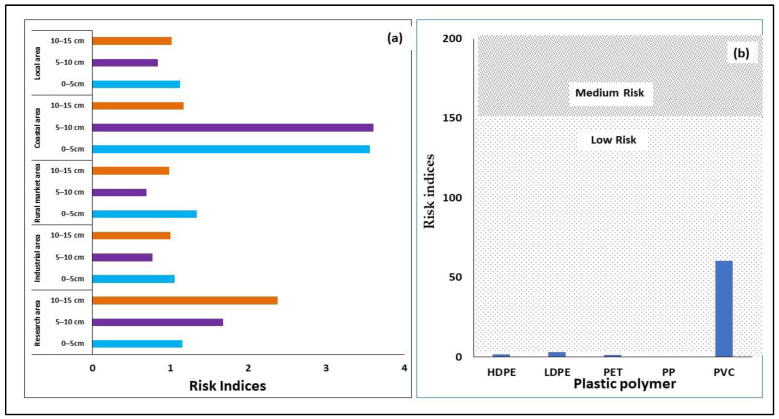
(**a**) Area-based risk indices of MPs; (**b**) individual risk indices of MPs in agricultural soils of Bangladesh.

The study assessed microplastic (MP) polymer risk indices (pRi) and overall pollution risk indices, with classifications by [57] ranging from low to very high based on pRi values. The results indicated low risk indices values ranging from 0.95 to 2.78 across all soil samples, suggesting low risks for all types of MPs (Figure 8a). Specifically, PVC exhibited comparatively higher health risk within the low-risk level (Figure 8b), with vinyl-chloride-containing plastics often considered toxic due to adverse health effects such as immune system disruption, hormonal imbalances, drowsiness, dizziness, headache, and liver impairment according to [33]. The prevalence of high-risk MPs in agricultural soils poses a potential health hazard for the farming community. These results align with the findings of [58], who reported low health risks of MPs in dust in Dhaka city, Bangladesh.

#### 3.7.2. Estimated Daily Intake (EDI) of MPs

The MPs enter the human body through ingestion, inhalation, and dermal exposure during farming practices. The results of the daily intake were obtained through three different pathways, as mentioned in Table 3. The results revealed that the estimated daily intake was higher in adults than in children across all areas and through different pathways. However, [29,59] reported different findings, indicating higher estimated daily intake (EDI) in children compared to adults. This difference may be attributed to the farming community being predominantly composed of adults in the context of Bangladesh, leading to higher EDI in this demographic group. Among the pathways, ingestion showed the highest estimated daily intake, followed by inhalation and dermal exposure. Additionally, the highest EDI was determined in the industrial area for both children and adults, followed by the research area, coastal area, rural area, and local market area, respectively. The industrial area exhibited a higher number of MPs compared to other areas. The highest EDI was recorded from PET microplastics in both children and adults in the industrial area, and from PP in the coastal and rural areas, while the lowest EDI was recorded from PP in the research area, industrial area, and local market area, and from HDPE in the coastal and rural areas. These findings are consistent with those of [29], who also reported the highest EDI of PET and the lowest EDI of PP.

#### 3.7.3. MP Carcinogenic Risk (MPCR) Assessment

The microplastic cancer risk (MPCR) of agricultural soils is detailed in Table 4, calculated using the cancer slope factor (CSF). According to the United States Environmental Protection Agency (USEPA), the standard limits for cancer risk range from 1 × 10^−6^ to 1 × 10^−4^ [33]. The results indicate that microplastics do not pose a carcinogenic risk, with the cancer risk value falling below the USEPA’s established limits. However, the highest risk was associated with PET in all areas except for the coastal region, where LDPE showed the highest risk. Moreover, farmers from industrial areas were found to face a higher risk compared to those from rural areas, with measured values exceeding the recommended dose. This risk assessment was based on detected microplastics in agricultural soil. Similar findings were reported by [58], who also observed cancer risk values lower than acceptable limits. It underscores the importance of comprehensive and detailed investigations to gather more consistent data for managing microplastic sources in agricultural soil and mitigating further contamination.

## 4. Conclusions

The analysis of microplastic (MP) concentration in agricultural soil revealed the presence of eight types of MPs: LDPE, HDPE, PET, PP, PVA, PVF, PVC, and PTFE. Organic manure and irrigation systems were identified as potential sources of LDPE and HDPE, whereas PET, PP, PVC, and their additives were associated with irrigation systems. MPs were observed in various shapes such as film, fiber, pellet, fragment, and irregular shapes, distributed across soil depths of 0–5 cm, 5–10 cm, and 10–15 cm. While the overall health risk from MPs in different areas was found to be low, individual MPs posed comparatively higher health risks, particularly PVC MPs. This study serves as a foundational investigation into the detection, characterization, source attribution, and probabilistic health risk assessment of MP contamination in various layers of non-mulched agricultural soils. Implementing precautionary measures to minimize MP contamination from different sources could significantly mitigate the entry of MPs into soil ecosystems.

## Figures and Tables

**Table 1:** General features of the selected areas of Bangladesh.

Parameters	Area 1	Area 2	Area 3	Area 4	Area 5
Area	Rural area	Local market area	Industrial Area	Coastal area	Research Area
Location	Thakurgaon	Joypurhat	Gazipur	Khulna	Barisal
Geographic coordinate	26.035505° N, 88.387421° E	25.054924° N, 89.012137° E	23.980278° N, 90.413611° E	22.738450° N, 89.590920° E	22.788271° N, 90.292958° E
Geographic attributes	Characterized by agricultural dominance, situated away from urban centers.	Adjacent to local markets with diverse grocery shops.	Located within industrial hubs, primarily housing garment industries, where waste management challenges persist due to a lack of awareness among the workforces. City corporation with insufficient waste management program.	Agricultural fields situated in coastal area, sharing characteristics influenced by unique coastal geography.	Represented by the Regional Station of Bangladesh Agricultural Research Institute (BARI), serving as a research environment restricted to the public.
Köppen–Geiger climate classification [27]	Equatorial monsoon (Am)	Equatorial monsoon (Am)	Equatorial monsoon (Am)	Equatorial monsoon (Am)	Equatorial monsoon (Am)
Soil type [28]	Acrisols, Luvisols	Acrisols, Luvisols	Acrisols, Gleysols	Solonchaks, Acrisols, Luvisols	Acrisols, Gleysols, Luvisols
Texture [28]	Clayey soil, Loamy	Clayey soil, Loamy	Loamy, Clayey soil	Clayey soil, Sandy to Loamy soil	Clayey soil, Loamy
Hydrogen (%)	0.17	0.24	0.23	0.55	0.45
Nitrogen (%)	0.1	0.11	0.13	0.18	0.18
Carbon (%)	0.83	1.16	1.45	1.73	2.52
CaCO_3_ (%)	5.92	5.55	5.62	8.91	7.78
Organic carbon (%)	0.12	0.49	0.78	0.66	1.59
Organic matter (%)	0.21	0.85	1.34	1.14	2.73
pH	6.1	5.7	6.5	8.5	7.5

**Table 2:** Parameters for the determination of health risk assessment of agricultural soils.

Parameters	Definition	Value for Children	Value for Adults	References
Exposure Duration (ED)	Exposure duration is the period an individual is exposed to a particular environmental condition, critically influencing the potential health effects and risks associated with that exposure. It is expressed as year (y).	6	30	[31]
Exposure Frequency (EF)	Exposure frequency is the number of times an individual is exposed to a particular environmental condition within a specific time. It is expressed as day/year (d/y).	180	180	
Average Time (AT) (Cancer)	Average time refers to the average number of days an individual is exposed to an agent, used in risk assessments over a specified period. AT Cancer = Lifetime (LT) × 365 = 76 × 365 = 27,740 d (Both) AT Non-cancer = ED × 365 = 6 × 365 = 2190 d (Children) AT Non-cancer = ED × 365 = 30 × 365 = 10,950 d (Adult)	27,740	27,740	
Average Time (AT) (Non- cancer)		2190	10,950	
Average Body Weight (BW)	Average body weight refers to the mean weight of an individual, measured in grams, and is often used in health-related studies to standardize dosages, exposures, and physiological assessments.	16,200	61,800	[32]
Average Lifetime (LT)	Average lifetime refers to the average number of years an individual is expected to live, used in various fields such as risk assessment to estimate long-term effects. It is expressed as a year.	76	76	
Particle Emission Factor (PEF)	The particle emission factor refers to the volume of air (in cubic meters) that contains one gram of emitted particles to quantify and model the release of particulate matter from various sources.	1.36 × 10^6^	1.36 × 10^6^	[33]
Ingestion Rate (IngR)	The ingestion rate refers to the amount of a substance consumed per day, measured in grams, and is used in dietary and exposure assessments to estimate the daily intake of MPs.	0.2	0.1	
Exposed Skin Area (SA)	Exposed skin area (SA) denotes the surface of the skin not shielded by clothing, crucial in evaluating risks from environmental factors and implementing appropriate safety measures. Its unit is cm^2^.	2800	5700	
Skin Adherence Factor (SL)	Skin adherence factor is a measure used to quantify the extent to which a substance adheres to the skin after application, influencing its absorption and potential systemic effects. It is expressed as mg/(cm^−2^·d^−1^).	0.2	0.07	
Inhalation Rate (InhR)	The inhalation rate refers to the volume of air an individual breathes in per day, measured in cubic meters, and is used in exposure assessments to estimate the intake of airborne MPs.	7.6	20	[34]
MP (Items/g)	Microplastic quantity noted as MP during calculation.			This study

**Table 3:** Estimated daily intake (particles/g/day) of microplastics through ingestion, inhalation, and dermal pathways.

Plastic Polymer	Pathways	Research Area		Industrial Area		Local Market Area		Coastal Area		Rural Area	
		Child	Adults	Child	Adults	Child	Adults	Child	Adults	Child	Adults
HDPE	Ingestion	3.91 × 10^−4^	3.73 × 10^−3^	3.55 × 10^−4^	3.39 × 10^−3^	2.84 × 10^−4^	2.71 × 10^−3^	3.55 × 10^−4^	3.39 × 10^−3^	7.10 × 10^−5^	6.80 × 10^−4^
	Inhalation	4.00 × 10^−11^	1.40 × 10^−10^	3.80 × 10^−11^	1.30 × 10^−10^	3.00 × 10^−11^	1.04 × 10^−10^	3.80 × 10^−11^	1.30 × 10^−10^	8.00 × 10^−12^	2.60 × 10^−11^
	Dermal	3.00 × 10^−11^	3.90 × 10^−11^	2.70 × 10^−11^	4.00 × 10^−11^	2.00 × 10^−11^	2.83 × 10^−11^	3.00 × 10^−11^	3.50 × 10^−11^	5.00 × 10^−12^	7.00 × 10^−12^
LDPE	Ingestion	4.26 × 10^−4^	4.06 × 10^−3^	7.46 × 10^−4^	7.11 × 10^−3^	3.91 × 10^−4^	3.73 × 10^−3^	6.39 × 10^−4^	6.10 × 10^−3^	4.26 × 10^−4^	4.06 × 10^−3^
	Inhalation	5.00 × 10^−11^	1.60 × 10^−10^	7.90 × 10^−11^	2.70 × 10^−10^	4.20 × 10^−11^	1.43 × 10^−10^	6.80 × 10^−11^	2.30 × 10^−10^	4.50 × 10^−11^	1.56 × 10^−10^
	Dermal	3.20 × 10^−11^	4.20 × 10^−11^	5.70 × 10^−11^	7.00 × 10^−11^	3.00 × 10^−11^	3.89 × 10^−11^	5.00 × 10^−11^	6.40 × 10^−11^	3.20 × 10^−11^	4.20 × 10^−11^
PET	Ingestion	8.88 × 10^−4^	8.47 × 10^−3^	1.03 × 10^−3^	9.82 × 10^−3^	4.97 × 10^−4^	4.74 × 10^−3^	5.68 × 10^−4^	5.42 × 10^−3^	4.62 × 10^−4^	4.40 × 10^−3^
	Inhalation	9.00 × 10^−11^	3.30 × 10^−10^	1.10 × 10^−10^	3.80 × 10^−10^	5.30 × 10^−11^	1.83 × 10^−10^	6.00 × 10^−11^	2.10 × 10^−10^	4.90 × 10^−11^	1.70 × 10^−10^
	Dermal	6.70 × 10^−11^	8.80 × 10^−11^	7.80 × 10^−11^	1.00 × 10^−10^	4.00 × 10^−11^	4.95 × 10^−11^	4.00 × 10^−11^	5.70 × 10^−11^	3.50 × 10^−11^	4.60 × 10^−11^
PP	Ingestion	2.49 × 10^−4^	2.37 × 10^−3^	2.49 × 10^−4^	2.37 × 10^−3^	1.78 × 10^−4^	1.69 × 10^−3^	7.46 × 10^−4^	7.11 × 10^−3^	5.68 × 10^−4^	5.42 × 10^−3^
	Inhalation	3.00 × 10^−11^	9.00 × 10^−11^	2.60 × 10^−11^	9.00 × 10^−11^	1.90 × 10^−11^	6.50 × 10^−11^	7.90 × 10^−11^	2.70 × 10^−10^	6.00 × 10^−11^	2.09 × 10^−10^
	Dermal	1.90 × 10^−11^	2.50 × 10^−11^	1.90 × 10^−11^	2.00 × 10^−11^	1.00 × 10^−11^	1.77 × 10^−11^	6.00 × 10^−11^	7.40 × 10^−11^	4.30 × 10^−11^	5.70 × 10^−11^
PVC	Ingestion	0	0	0	0	0	0	6.77 × 10^−4^	6.80 × 10^−4^	0	0
	Inhalation	0	0	0	0	0	0	8.00 × 10^−12^	3.00 × 10^−11^	0	0
	Dermal	0	0	0	0	0	0	1.00 × 10^−11^	7.00 × 10^−12^	0	0

**Table 4:** Microplastics carcinogenic risk index.

Plastic Polymer	Research Area	Industrial Area	Local Market Area	Coastal Area	Rural Area
HDPE	2.61 × 10^−10^	2.37 × 10^−10^	1.9 × 10^−10^	2.37 × 10^−10^	4.75 × 10^−11^
LDPE	2.85 × 10^−10^	4.97 × 10^−10^	2.61 × 10^−10^	4.27 × 10^−10^	2.85 × 10^−10^
PET	5.94 × 10^−10^	6.86 × 10^−10^	3.32 × 10^−10^	3.8 × 10^−10^	3.09 × 10^−10^
PP	3.91 × 10^−11^	3.9 × 10^−11^	2.79 × 10^−11^	1.17 × 10^−10^	8.94 × 10^−11^
PVC	0	0	0	8.85 × 10^−11^	0
Total	1.18 × 10^−9^	1.46 × 10^−9^	8.12 × 10^−10^	1.25 × 10^−9^	7.31 × 10^−10^

## Data Availability

The data that support the findings of this study are available on request from the corresponding author.
